# Clinicopathologic features and prognostic grouping of gastrointestinal stromal tumors (GISTs) in Pakistani patients: an institutional perspective

**DOI:** 10.1186/s13104-018-3562-8

**Published:** 2018-07-11

**Authors:** Atif Ali Hashmi, Mahrukh Faraz, Zareeha Nauman, Muhammad Usman Qureshi, Shumaila Kanwal Hashmi, Hira Fatima Waseem, Muhammad Muzzammil Edhi, Naveen Faridi, Amir Khan

**Affiliations:** 1 Department of Pathology, Liaquat National Hospital and Medical College, Karachi, Pakistan; 2 Department of Pathology, Aga Khan University, Karachi, Pakistan; 3 Department of Pathology, CMH Institute of Medical Sciences, Multan, Pakistan; 4 Department of Surgery, Brown University, Providence, RI USA; 5 Department of Medicine, Kandahar University, Kandahar, Afghanistan

**Keywords:** Gastrointestinal stromal tumors, GISTs, Epitheloid GIST, Spindle cell GIST

## Abstract

**Objectives:**

Gastrointestinal stromal tumors (GISTs) are rare tumors of gastrointestinal tract, prognosis of which largely depends upon histopathologic characteristics of resection specimens, which were not widely studied in our population. Therefore we aimed to evaluate the histopathologic characteristics of GISTs in our population and their prognostic grouping according to college of American pathologist’s guidelines.

**Results:**

Mean age of patients was 53.4 years (18–71 years). 92% of cases were of primary GISTs and stomach was the most common site (57.7%). 75% of cases were of spindle cell morphology and 53.8% belonged to high risk prognostic group. Comparison of stomach and intestinal GISTs showed that intestinal GISTs were found to be of high grade (70%) and of high risk prognostic group (75 and 80%) compared to stomach GISTs (43% were of high risk prognostic group), however this finding was not statistically significant. GISTs are infrequent gastrointestinal tumors but early diagnosis and identification of adverse histological features are key to successful treatment. We found a large majority of GISTs to be located in stomach, however intestinal GISTs were found more likely to be associated with adverse prognostic parameters. However more large scale studies are warranted to establish this finding.

**Electronic supplementary material:**

The online version of this article (10.1186/s13104-018-3562-8) contains supplementary material, which is available to authorized users.

## Introduction

Gastrointestinal stromal tumors (GISTs) are the most common mesenchymal tumors of the gut, however overall they still account for < 1% of all gastrointestinal tumors [1]. The clinical signs and symptoms of GISTs are non-specific abdominal discomfort and distention, therefore the diagnosis and treatment is usually delayed leading to therapy failures and high morbidity and mortality rates. The incidence of GISTs at present is about 15 cases in 1 million in the United States and about 11 cases in one million in Northern Europe. Although, the incidence of GISTs before 2000 is unknown; but the growing number of presenting cases has led to increased research about this subject [2, 3]. The incidence of GISTs in our country is unknown as large scale studies have not been conducted.

Morphologically, the features of GIST resemble that of leiomyoma and leiomyosarcoma and were previously classified as such [4]. Almost all recent researches have reached to the conclusion that GISTs can occur anywhere throughout the digestive tract but most number of GIST cases were recorded in the stomach [5].

The pathologic parameters of GISTs in resection specimens are important in guiding post-operative management and determining prognosis of the patients, however these features have not been widely studied in our population. Only a few studies have been conducted in Pakistan. Ud Din et al. evaluated 255 cases of GIST and found 62.3 gastric, 81.8% duodenal, 68% small intestinal, 72% colorectal and 89% GISTs to be of high risk category [6]. Similarly Mushtaq et al. performed risk stratification on 36 cases of GIST. They found seven patients to fall into low risk, ten patients intermediate risk, and 19 patients in high risk groups. There were no patients in very low risk group [7]. Therefore in this study we aimed to evaluate clinicopathologic and prognostic parameters of GISTs in our population which can help in devising personalized therapeutic regimens for loco-regional population.

## Main text

### Materials and methods

A total of 52 cases of GISTs diagnosed and treated at Liaquat National hospital were included in the study from 2011 till 2016. An approval from institutional ethical review committee was taken antecedent to conducting the study. All cases were biopsy proven prior to definite resection. After pre-operative workup, definite resection was performed and specimens were sent to the pathology department. Gross and microscopic features of all tumors were recorded including tumor size, site, tumor morphology, grade, number of mitosis and prognostic group according to College of American Pathologists (CAP) guidelines.

### Immunohistochemistry

Immunohistochemical markers including CKAE1/3, ASMA, S100, CD34 and CD117 were performed by DAKO envision method and slides were interpreted by experienced pathologists. For CD117 IHC, polyclonal Rabbit anti-human CD117, c-kit antibody was used purchased from DAKO and IHC was performed according to DAKO envision method. Moderate to strong membranous staining in more than 10% tumor cells is considered positive. For CD34, FLEX monoclonal anti-human CD34 class II, clone QBEnd 10, ready to use antibody was used. For S100 IHC, FLEX polyclonal rabbit Anti-S100 ready to use antibody was purchased from DAKO. Similarly, for ASMA IHC, monoclonal anti-human Smooth muscle actin, clone 1A4 antibody was used and performed using DAKO envision kit according to manufacturer’s recommendations. Moderate to strong cytoplasmic staining in more than 10% tumor cells was considered positive for ASMA, S100 and CD34.

### Statistical analysis

Statistical package for social sciences (SPSS 21) was used for data compilation and analysis. Mean and standard deviation were calculated for quantitative variables. Frequency and percentage were calculated for qualitative variables. Chi square was applied to determine association. P-value of ≤ 0.05 was considered as significant.

### Results

**Fig. 1:**
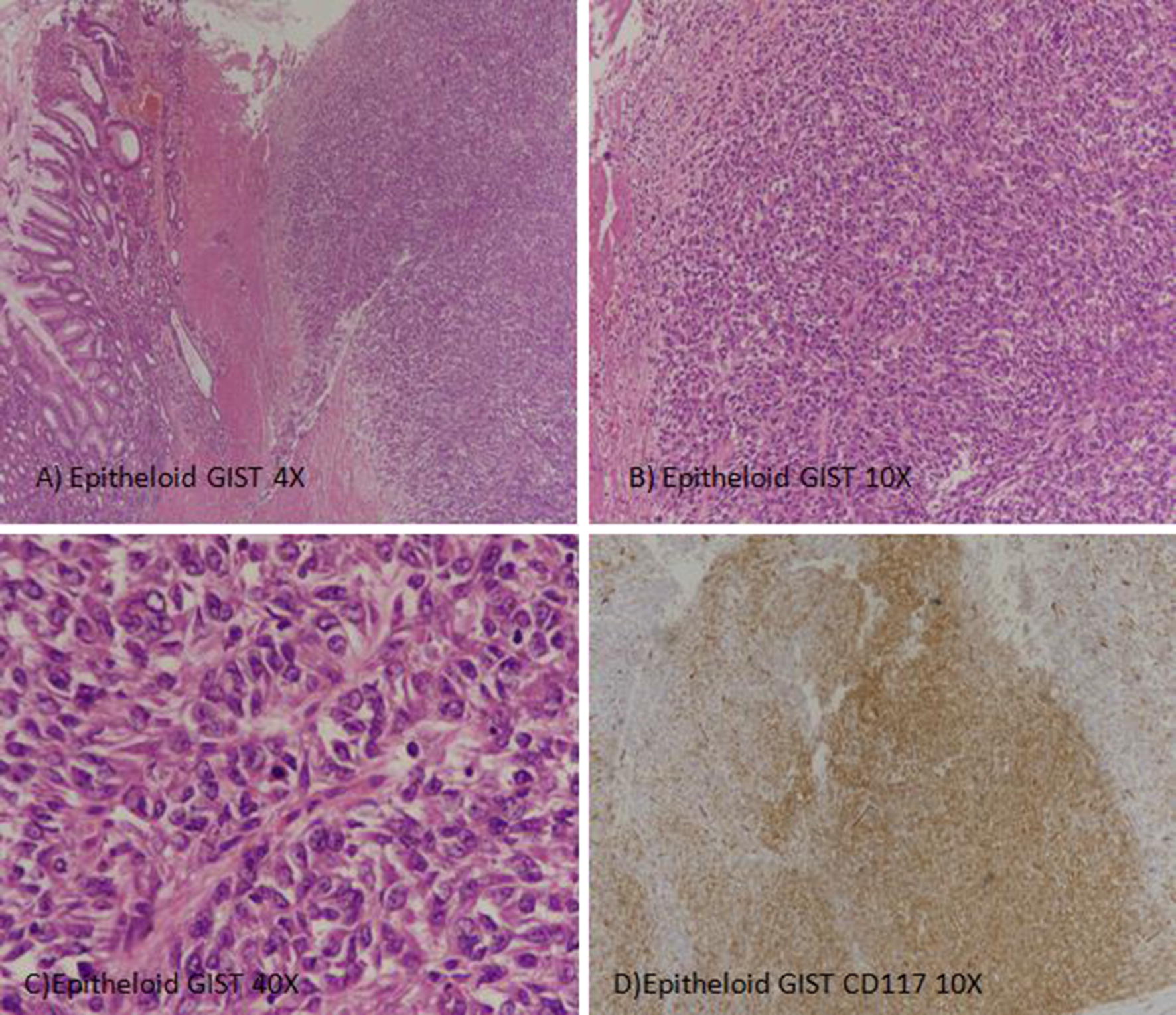
Gastrointestinal stromal tumor, Epitheloid subtype: **A**–**C** H&E sections showing sheets of epitheloid cells with marked atypia. **D** Diffuse expression of CD117 noted in tumor cells

Mean age of patients was 53.4 years (18–71 years) with a slight male predominance. 92% of cases were of primary GISTs and stomach was the most common site (57.7%). 75% of cases were of spindle cell morphology and 53.8% belonged to high risk prognostic group as shown in Table 1. Table 2 shows the comparison of GISTs at various sites of digestive tract. Out of 48 cases of primary GISTs, 30 cases were seen in stomach, 10 in small intestine and 8 in large intestine. Out of 30 GISTs of stomach, 20 were in the age group of > 50 years, 9 were seen in the age group of 31–50 years and only 1 case of stomach GIST was seen in age group of < 30 years. Similarly, in the small and large intestine, the larger number of cases were seen in the age group of > 50 years. The gender predominance was not much appreciated, as equal number of cases of stomach GIST was seen in both male and female. However, the small and large intestines GISTs were seen to be more common in males, however this finding was not statistically significant. Majority of the tumors were greater than 10 cm in size, however most GISTs in the stomach were found to be 5–10 cm in size and most GISTs in the small and large intestine were greater than 10 cm in size. 37 out of 48 cases were of spindle cell morphology. 20 out of 30 cases of stomach GIST were of spindle cell morphology, while 3 were epitheloid (Fig. 1) and 7 were of mixed morphology. Almost all cases of small and large intestine GIST were of spindle cell variety (Additional file 1: Figure S1). 27 cases fell in the category of high risk category, 5 in the moderate risk, 11 in the low risk and 5 in the very low risk. Majority of the cases displayed a mitotic activity of greater than 5/50 HPF as a whole. 34 out of 46 tumors were CD34 positive and 46 out of 48 were CD117 positive. 12 out of 40 were positive for S100 and 19 out of 43 were positive for ASMA. Hence, majority of the tumors were positive for CD34 and CD117 and negative for S100 and ASMA as shown in Table 3.

**Table 1:** Clinicopathologic characteristics of gastrointestinal tumors (GISTs)

Characteristic	Frequency (%)
Gender	
Male	32 (61.5%)
Female	20 (38.51%)
Age (years)	
Mean	53.4 (18–71 years)
Age groups (years)	
< 30	3 (5.8%)
31–50	18 (34.61%)
> 50	31 (59.6%)
Primary/metastatic	
Primary	48 (92.3%)
Metastasis	4 (7.74%)
Site	
Stomach	30 (57.7%)
Small intestine	10 (19.2%)
Large intestine	08 (15.4%)
Liver	04 (7.7%)
Size (cm)	
Mean	9.4 (2–16)
Size groups (cm)	
< 2	1 (1.9%)
2–5	6 (11.5%)
5–10	20 (35.5%)
> 10	22 (42.3%)
Morphology	
Spindle	39 (75%)
Eptheloid	4 (7.5%)
Mixed	9 (17.3%)
Grade (mitotic activity)	
Low grade (< 5/50HPFs)	23 (44.2%)
High grade (> 5/50HPFs)	29 (55.8%)
Necrosis	
Present	10 (19.2%)
Absent	42 (80.8%)
Prognostic groups	
Very low risk	5 (9.63%)
Low risk	11 (21.2%)
Moderate risk	5 (9.6%)
High risk	28 (53.8%)

**Table 2:** Comparison of clinicopathologic features of gastrointestinal stromal tumors (GISTs) of various sites

Variables	Stomach	Small intestine	Large intestine	P-value
	N (%)	N (%)	N (%)	
Age (years)				
Mean ± SD	54.50 ± 11.99	52.10 ± 15.53	55.25 ± 14.29	0.852
Age groups (years)				
≤ 30	1 (3.3)	1 (10)	0	0.785
31–50	9 (30)	4 (40)	3 (37.5)	
> 50	20 (66.7)	5 (50)	5 (62.5)	
Gender				
Male	15 (50)	8 (80)	6 (75)	0.159
Female	15 (50)	2 (20)	2 (25)	
Size				
Mean ± SD	8.96 ± 3.94	10.20 ± 4.75	10.28 ± 3.11	0.570
Size groups (cm)				
≤ 2	0	1 (10)	0	0.287
2.1–5	4 (13.3)	1 (10)	1 (12.5)	
5.1–10	15 (50)	2 (20)	2 (25)	
> 10	11 (36.7)	6 (60)	5 (62.5)	
Morphology				
Spindle cell	20 (66.7)	10 (100)	7 (87.5)	0.318
Eptheloid	3 (10)	0	0	
Mixed	7 (23.3)	0	1 (12.5)	
Prognostic group				
Very low risk	4 (13.3)	1 (10)	0	0.214
Low risk	10 (33.3)	0	1 (12.5)	
Moderate risk	3 (10)	1 (10)	1 (12.5)	
High risk	13 (43.3)	8 (80)	6 (75)	
Grade (mitotic activity)				
Low grade (≤ 5/50HPFs)	15 (50)	3 (30)	5 (62.5)	0.456
High grade (> 5/50HPFs)	15 (50)	7 (70)	3 (37.5)

**Table 3:** Immunohistochemal features of gastrointestinal tumors (GISTs)

	S100				ASMA				CD34				CD117		
	P (n = 12)	N (n = 28)	ND (n = 8)	P-value	P (n = 19)	N (n = 24)	ND (n = 5)	P-value	P (n = 34)	N (n = 12)	ND (n = 2)	P-value	P (n = 46)	N (n = 2)	P-value
Site	7 (58.3)	18 (64.3)	5 (62.5)	0.515	10 (52.6)	17 (70.8)	3 (60.0)	0.469	25 (73.5)	3 (25.0)	2 (100)	0.013*	28 (60.9)	2 (100)	1.000
Stomach (n = 30)	3 (25.0)	4 (14.3)	3 (37.5)		4 (21.1)	4 (16.7)	2 (40.0)		6 (17.6)	4 (33.3)	0		10 (21.7)	0	
Small intestine (n = 10)	2 (16.7)	6 (21.4)	0		5 (26.3)	3 (12.5)	0		3 (8.8)	5 (41.7)	0		8 (17.4)	0	
Large intestine (n = 8)															
Morphology															
Spindle cell (n = 37)	9 (75)	21 (75.0)	7 (87.5)	1.000	12 (63.2)	20 (83.3)	5 (100)	0.462	25 (73.5)	10 (83.3)	2 (100)	0.137	37 (80.4)	0	0.024*
Eptheloid (n = 3)	1 (8.3)	2 (7.1)	0		2 (10.5)	1 (4.2)	0		1 (2.9)	2 (16.7)	0		2 (4.3)	1 (50.0)	
Mixed (n = 8)	2 (16.7)	5 (17.9)	1 (12.5)		5 (26.3)	3 (12.5)	0		8 (23.5)	0	0		7 (15.2)	1 (50.0)	
Grade															
Low (n = 29)	5 (41.7)	17 (60.7)	7 (87.5)	0.123	11 (57.9)	13 (54.2)	5 (100)	0.220	23 (67.6)	5 (41.7)	1 (50.0)	0.263	29 (63.0)	0	0.152
High (n = 19)	7 (58.3)	11 (39.3)	1 (12.5)		8 (42.1)	11 (45.8)	0		11 (32.4)	7 (58.3)	1 (50.0)		17 (37.0)	2 (100.0)	
Prognostic group															
Very low risk (n = 5)	0	4 (14.3)	1 (12.5)	0.668	1 (5.3)	3 (12.5)	1 (20)	0.681	4 (11.8)	0	1 (50.0)	0.190	5 (10.9)	0	1.000
Low risk (n = 11)	2 (16.7)	6 (21.4)	3 (37.5)		6 (31.6)	4 (16.7)	1 (20)		10 (29.4)	1 (8.3)	0		11 (23.9)	0	
Moderate risk (n = 5)	1 (8.3)	3 (10.7)	1 (12.5)		2 (10.5)	2 (8.3)	1 (20)		4 (11.8)	1 (8.3)	0		5 (10.9)	0	
High risk (n = 27)	9 (75.0)	15 (53.6)	3 (37.5)		10 (52.6)	15 (62.5)	2 (40)		16 (47.1)	10 (83.3)	1 (50.0)		25 (54.3)	2 (100)	

*P* represents positive, *N* represents negative, *ND* represents not done

* Significant at 0.05 level

### Discussion/conclusion

GISTs have long been known to be mesenchymal tumors of the gastrointestinal tract [2, 8]. Historically, they were considered rare tumors mainly due to the reason that they were mostly misdiagnosed owing to the similarities they shares with leiomyomas, leimyosarcomas and schwannomas [9]. The misdiagnosis led to a bad prognosis and treatment failures. However, the attempts made in the recent years to better understand the occurrence, incidence and morphology of GIST has established the fact that they are the most common mesenchymal tumor of the GIT [1]. They can occur anywhere along the length of the GIT, most common location of GIST occurrence being the stomach [5].

In this study, we specifically compared the general characteristics of GIST with respect to the location and the histochemical markers (as they have proved to be in an essential tool for the diagnosis of GIST) and compared them with previously published literature.

Although most of the literature quotes the presence of GIST in esophagus, stomach, intestine, rectum and mesentery; in this study of 48 cases of primary GIST, the occurrence was seen in stomach, small intestine and large intestine only. As per previous studies, stomach predominated with 62.5% followed by small intestine (20.8%) and large intestine (16.7%). This was consistent with the findings of most of the other Asian literature.

GISTs were seen to be more common in the older age adults of greater than 50 years and very rarely seen in young adults of less than 30 years. Some cases were also seen in the age group of 30–50 but it was not so commonly seen in this age group, mean age of stomach GIST being 54.50, 52.10 of small intestine and 55.25 of large intestine. Although not statistically significant (P = 0.785) but in accordance with other studies done, we can say that GIST is most likely to occur in older age adults of greater than 50 years [10–12].

Male and female genders were equally affected by stomach GIST (50% cases were reported in both), however the intestinal GIST were predominantly seen in males than females (80% vs 20% in small intestine and 75% vs 25% in large intestine). Although other Asian studies did show slight male to female dominance [10, 11, 13], in this study no statistical significance was seen (P = 0.159).

GISTs usually involve the entire thickness of the gastrointestinal wall [14, 15], this owes to the fact that they are usually larger in size, as established in this study where majority of the tumors were greater than 10 cm and scarcely less than 5 cm. The reason behind the large size of the tumor might be its relatively silent clinical course [10]. However, the mean size of the tumor in the stomach was seen to be 8.96 and 10.20 and 10.28 in the small and large intestine respectively. Although, not statistically significant (P = 0.570) it can be noticed that majority of the tumors in the stomach ranged from 5 to 10 cm in size and majority of the intestinal tumors were greater than 10 cm. Some other Asian studies have also mentioned the mean size of the tumor to be > 5 cm [13, 16].

On histology, the majority tumors composed of spindle cells (77%) arranged in interlacing pattern forming whorls, with abundant eosinophilic cytoplasm. Epitheloid and mixed varieties were rarely seen, however among these two, the mixed variety predominated (10% and 35.8% respectively); although epitheloid type has been mentioned to be more common than mixed in the previous literatures [15] but our finding was consistent with the findings of Asian literature in which mixed variety predominated [10, 11]. Nonetheless, spindle variety was most common finding in all studies. Although not statistically significant, but it was noted that the stomach contained all three types of morphology patterns while 100% of the cases of the small intestinal GISTs were of spindle cell morphology and the large intestinal GISTS were seen to have spindle and mixed morphology (87.5 and 12.5% respectively).

According to Asian studies, most of the GISTs overall were low grade tumors [11] and most showed high risk features followed by intermediate and low risk [12, 13, 17]. In our study, majority of the stomach GISTs were seen to be of high risk (43.3%), followed by low risk, very low risk and moderate risk. However, the greatest high risk tumors were in the small intestine (80%) and large intestine (75%).

For the purpose of studying the immunohistochemical features of the GISTs, two types of antibodies were used: one with high specificity for GISTs, such as CD117 and CD34, and other which are more specific for smooth muscle tumors and neural tumors (ASMA and S-100), as these two types of tumors are the ones which are most often misdiagnosed as GISTs.

S-100 was positive in 12 cases, negative in 28, not performed in 8. ASMA was positive in 19 cases, negative in 24 and not performed in 5. CD34 was positive in 34 cases, negative in 12 and not performed in 2. CD117 was positive in 46 cases and negative in 2. These findings are consistent with many other Asian studies in which CD117 and CD34 positivity has been seen in most GISTs, followed by ASMA and S-100 [10, 13, 16, 18].

While, most of the stomach GISTs were negative for ASMA (70.8%) and S-100 (64.3%) and positive for CD34 and CD117 (as well as most of small intestine tumors were positive for both), most of the large intestine GISTs were seen to be positive for ASMA (26.3%) and negative for CD34 (41.7%). Most of the spindle cell variety was negative for ASMA (83.3%) and positive for CD34 (73.5%) and most of the epitheloid and mixed variety were positive for ASMA (10.5 and 26.3%) and CD34 negativity was noticed in most epitheloid type variety. Most of the high risk tumors were negative (62.5%) and low risk tumors were positive (31.6%) for ASMA.

A statistically significant finding was seen in CD34 positivity with respect to site of the tumor (P = 0.013) and CD117 positivity with respect to the morphology of the tumor (P = 0.024); other findings however, were not statistically significant.

Liu et al. compared 300 cases of duodenal GISTs with gastric GISTs and found them to be significantly associated with worse prognostic features [19]. Similarly Zhu et al. compared colorectal GISTs with gastric GISTs. They found rectal GISTs to be associated with improved overall survival while colonic GISTs were associated with worse overall survival [20]. On the other hand Feng et al. studied small intestinal GISTs and found jejunal and ileal GISTs to be comparable in terms of prognosis [21].

## Limitations

GISTs are infrequent gastrointestinal tumors but early diagnosis and identification of adverse histological features are key to successful treatment. We found a large majority of GISTs to be located in stomach, however intestinal GISTs were found more likely to be associated with adverse prognostic parameters. One of the major limitations of the study was small sample size and lack of clinical follow up to determine disease free survival and recurrence. Therefore we suggest that, more large scale studies are warranted to establish the findings of our study.

## Additional file

**Additional file 1: Figure S1.** Gastrointestinal tumor, spindle cell subtype: (A, B) H&E sections showing sheets of spindled cells with elongated nuclei. C, D Tumor cells show CD117 and CD34 positivity.

## Abbreviations

GISTs: gastrointestinal stromal tumors
CAP: College of American Pathologists

## References

[1] Fülöp E, Marcu S, Milutin D, Borda A (2009). Gastrointestinal stromal tumors: review on morphology, diagnosis and management. Rom J Morphol Embryol.

[2] Nilsson B, Bümming P, Meis-Kindblom JM, Odén A, Dortok A, Gustavsson B, Sablinska K, Kindblom LG (2005). Gastrointestinal stromal tumors: the incidence, prevalence, clinical course, and prognostication in the preimatinib mesylate era—a population-based study in western Sweden. Cancer.

[3] Tryggvason G, Gíslason HG, Magnússon MK, Jónasson JG (2005). Gastrointestinal stromal tumors in Iceland, 1990–2003: the Icelandic GIST study, a population-based incidence and pathologic risk stratification study. Int J Cancer.

[4] Bucher P, Villiger P, Egger JF, Buhler LH, Morel P (2004). Management of gastrointestinal stromal tumors: from diagnosis to treatment. Swiss Med Wkly..

[5] Miettinen M, Majidi M, Lasota J (2002). Pathology and diagnostic criteria of gastrointestinal stromal tumors (GISTs): a review. Eur J Cancer.

[6] Ud Din N, Ahmad Z, Arshad H, Idrees R, Kayani N (2015). Gastrointestinal stromal tumors: a clinicopathologic and risk stratification study of 255 cases from Pakistan and review of literature. Asian Pac J Cancer Prev.

[7] Mushtaq S, Mamoon N, Hassan U, Iqbal M, Khadim MT, Sarfraz T (2009). Gastrointestinal stromal tumors-a morphological and immunohistochemical study. J Gastrointest Cancer..

[8] Duensing A, Heinrich MC, Fletcher CD, Fletcher JA (2004). Biology of gastrointestinal stromal tumors: KIT mutations and beyond. Cancer Invest.

[9] Liu P, Na J, Wang Y, He Q, Zhang Y, Tang X, Zou W (2002). Study of gastrointestinal stromal tumors by light microscopy, electron microscopy and immunohistochemistry. Zhonghua Binglixue Zazhi..

[10] Hou Y, Wang J, Zhu X, Du X, Sun M, Zheng A, Bing Z, Xue L, Zhi Z (2002). A clinicopathologic and immunohistochemical study on 76 cases of gastrointestinal stromal tumors. Chin J Pathol.

[11] Hasegawa T, Matsuno Y, Shimoda T, Hirohashi S (2002). Gastrointestinal stromal tumor: consistent CD117 immunostaining for diagnosis, and prognostic classification based on tumor size and MIB-1 grade. Hum Pathol.

[12] Kapoor R, Khosla D, Kumar P, Kumar N, Bera A (2013). Five-year follow up of patients with gastrointestinal stromal tumor: recurrence-free survival by risk group. Asia Pac J Clin Oncol.

[13] Rauf F, Bhurgri Y, Pervez S (2007). Gastrointestinal stromal tumors: a demographic, morphologic and immunohistochemical study. Indian J Gastroenterol.

[14] Fenoglio-Preiser CM, Noffsinger AE, Stemmermann GN, Lantz PE, Isaacson PG (2008). Gastrointestinal pathology: an atlas and text. Mesenchymal tumors.

[15] Fülöp E, Marcu S, Borda A, Moldovan C, Fülöp EF, Loghin A, Pávai Z (2011). Histopathological and immunohistochemical features of gastrointestinal stromal tumors. Rom J Morphol Embryol.

[16] Liu FY, Qi JP, Xu FL, Wu AP (2006). Clinicopathological and immunohistochemical analysis of gastrointestinal stromal tumor. World J Gastroenterol.

[17] Kim MK, Lee JK, Park ET, Lee SH, Seol SY, Chung JM, Kang MS, Yoon HK (2004). Gastrointestinal stromal tumors: clinical, pathologic features and effectiveness of new diagnostic criteria. Korean J Gastroenterol.

[18] Ji F, Wang ZW, Wang LJ, Ning JW, Guo-Qiang X (2008). Clinicopathological characteristics of gastrointestinal mesenchymal tumors and diagnostic value of endoscopic ultrasonography. J Gastroenterol Hepatol.

[19] Liu Z, Zheng G, Liu J, Liu S, Xu G, Wang Q, Guo M, Lian X, Zhang H, Feng F (2018). Clinicopathological features, surgical strategy and prognosis of duodenal gastrointestinal stromal tumors: a series of 300 patients. BMC Cancer..

[20] Zhu R, Liu F, Grisotti G, Pérez-Irizarry J, Cha CH, Johnson CH, Boffa DJ, Han D, Johung KL, Zhang Y, Khan SA (2018). Distinctive features of gastrointestinal stromal tumors arising from the colon and rectum. J Gastrointest Oncol..

[21] Feng F, Wang F, Wang Q, Zheng G, Xu G, Liu S, Liu Z, Guo M, Lian X, Zhang H (2018). Clinicopathological features and prognosis of gastrointestinal stromal tumor located in the jejunum and ileum. Dig Surg..

