# Supplementary figures and images for: Clinicopathologic features and prognostic grouping of gastrointestinal stromal tumors (GISTs) in Pakistani patients: an institutional perspective
Source: BMC Res Notes. 2018 Jul 11;11:457. doi: 10.1186/s13104-018-3562-8 (PMC6042430; doi:10.1186/s13104-018-3562-8)

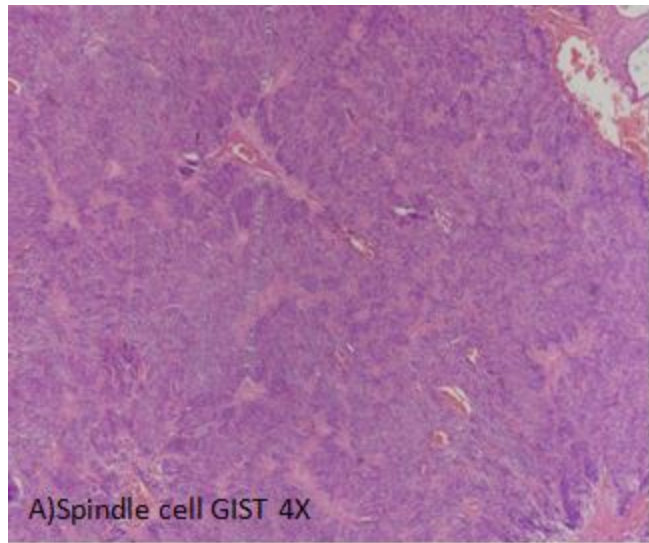

A)Spindle cell GIST 4X

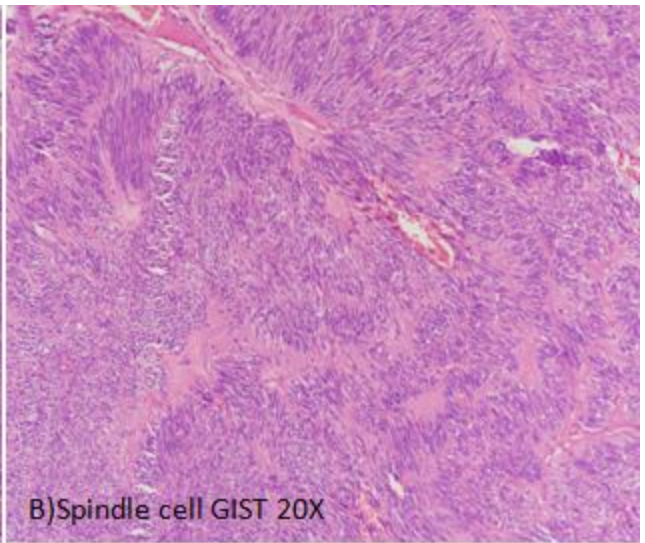

B)Spindle cell GIST 20X

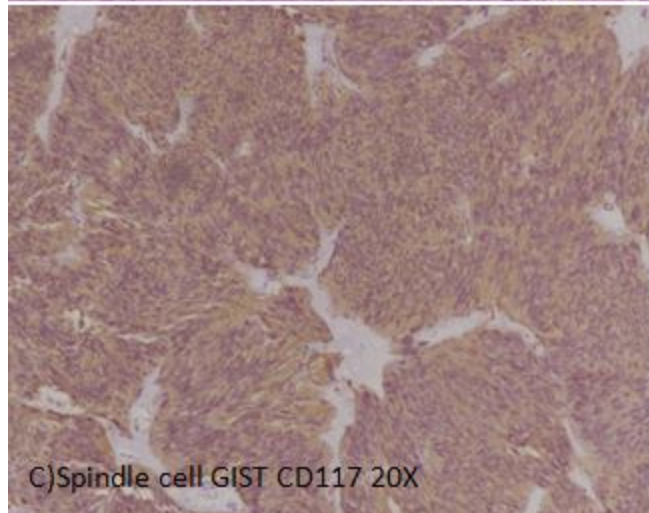

C)Spindle cell GIST CD117 20X

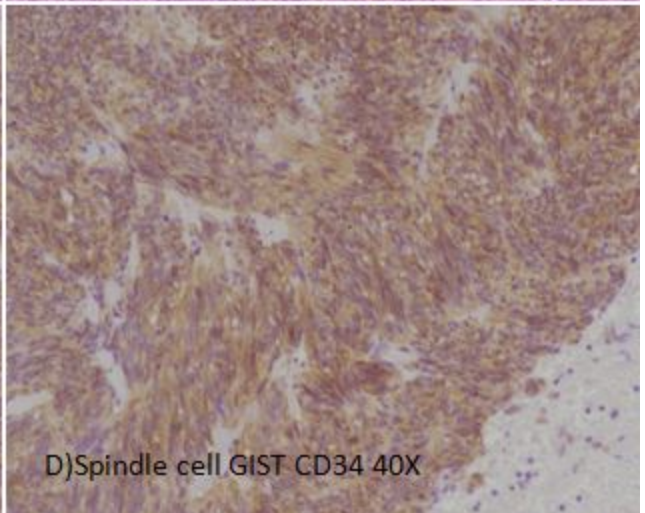

D)Spindle cell GIST CD34 40X

Supplement: Supplementary file 1 — Additional file 1: Figure S1. Gastrointestinal tumor, spindle cell subtype: (A, B) H&E sections showing sheets of spindled cells with elongated nuclei. C, D Tumor cells show CD117 and CD34 positivity. [file 13104_2018_3562_MOESM1_ESM.pdf]
